# Complete Genome Sequence of Equid Herpesvirus 3

**DOI:** 10.1128/genomeA.00797-14

**Published:** 2014-10-02

**Authors:** Steven Sijmons, Aldana Vissani, Maria Silva Tordoya, Benoît Muylkens, Etienne Thiry, Piet Maes, Jelle Matthijnssens, Maria Barrandeguy, Marc Van Ranst

**Affiliations:** a Laboratory of Clinical Virology, Rega Institute for Medical Research, KU Leuven, Leuven, Belgium; b Instituto de Virología, CICVyA, INTA-Castelar, Buenos Aires, Argentina; c Veterinary Virology and Animal Viral Diseases, Department of Infectious and Parasitic Diseases, Faculty of Veterinary Medicine, University of Liège, Liège, Belgium

## Abstract

Equid herpesvirus 3 (EHV-3) is a member of the subfamily *Alphaherpesvirinae* that causes equine coital exanthema. Here, we report the first complete genome sequence of EHV-3. The 151,601-nt genome encodes 76 distinct genes like other equine alphaherpesviruses, but genetically, EHV-3 is significantly more divergent.

## GENOME ANNOUNCEMENT

Equid herpesvirus 3 (EHV-3) belongs to the genus *Varicellovirus* of the subfamily *Alphaherpesvirinae*. EHV-3 is antigenically, genetically, and pathogenically distinct from the other equine herpesviruses and has a worldwide distribution. EHV-3 is the causative agent of equine coital exanthema (ECE). ECE is an infectious, venereally transmitted disease with characteristic papules, vesicles, pustules, and ulcers on the external genitalia of mares and stallions. Although EHV-3 is highly contagious, the virus is non-invasive, and ECE is a fairly benign disease. ECE, however, has a significant negative economic impact on the equine breeding industry due to forced temporary disruption of mating activities (1). Although the complete genomes of four equine alphaherpesviruses (EHV-1, EHV-4, EHV-8, and EHV-9) have been published (2–5), the sequence knowledge for EHV-3 is limited to fragments of the DNA polymerase, the DNA packaging protein, and the glycoprotein G (6–8). We therefore determined the complete genome sequence of EHV-3.

A perineal-vaginal swab was collected from a mare showing typical lesions of ECE. EHV-3 was isolated and multiplied on equine dermal cells (E. Derm, NBL-6) for two passages and plaque purified three times. Clone 3A was passaged seven additional times in the same cell line, and 600 mL of culture supernatant (TCID_50_ 10^5^) was clarified (30 min at 3,500 rpm), concentrated (1 h at 100,000 g), and purified with a 30% sucrose gradient ultracentrifugation (2 h at 100,000 g). Subsequently, residual equine DNA was degraded through nuclease digestion, after which viral DNA was extracted as previously described (9). Viral DNA was amplified using multiple displacement amplification (REPLI-g, Qiagen) and analyzed using 454 GS FLX (Roche) and MiSeq (Illumina) sequencing platforms. Sequence reads were mapped on the equine genome and unmapped reads were collected for further assembly (10, 11). *De novo* assemblies were conducted separately using 454 and Illumina datasets (with Newbler v2.6 and Velvet v0.7.56 [12], respectively), and contigs were merged using Phrap v1.090518. This resulted in 12 contigs that could be extended and joined by an iterative mapping of the sequence reads (MIRA v3.4.1.1). Finally, all sequence reads were mapped on the EHV-3 genome sequence, the assembly was visually inspected (13), and misassemblies were corrected. Remaining uncertainties were verified through PCR amplification and Sanger sequencing.

EHV-3 has a class D genome, consisting of a long and a short unique region (UL and US), both flanked by inverted repeats (TRL/IRL and IRS/TRS). It has a total size of 151,601 nt and a G+C content of 68.1%. EHV-3 open reading frames (ORFs) are arranged in the same order as in other equine alphaherpesviruses with no proof of additional ORFs. Due to a different position of the junction between US and IRS/TRS, ORF 76 is positioned inside the IRS/TRS and is duplicated. Consequently, EHV-3 is predicted to encode 76 distinct genes, 4 of which are duplicated (ORFs 64, 65, 66, and 76), resulting in a total of 80 genes. Genetically, EHV-3 is the most divergent of the equine alphaherpesviruses with overall nucleotide identities with the other viruses ranging from 62.1% to 64.9%, whereas EHV-1, EHV-4, EHV-8, and EHV-9 have identities of at least 78.2%.

### Nucleotide sequence accession number.

The genome sequence of EHV-3 has been deposited in GenBank under the accession number KM051845.
